# Nanomicelles of Radium Dichloride [^223^Ra]RaCl_2_ Co-Loaded with Radioactive Gold [^198^Au]Au Nanoparticles for Targeted Alpha–Beta Radionuclide Therapy of Osteosarcoma

**DOI:** 10.3390/polym14071405

**Published:** 2022-03-30

**Authors:** Bárbara Nayane Rosário Fernandes Souza, Elisabete Regina Fernandes Ramos Ribeiro, Aline Oliveira da Silva de Barros, Martha Sahylí Ortega Pijeira, Hericka Oliveira Kenup-Hernandes, Eduardo Ricci-Junior, Joel Félix Silva Diniz Filho, Clenilton Costa dos Santos, Luciana Magalhães Rebelo Alencar, Mohamed F. Attia, Sara Gemini-Piperni, Ralph Santos-Oliveira

**Affiliations:** 1 Argonauta Nuclear Reactor Center, Nuclear Engineering Institute, Brazilian Nuclear Energy Commission, Rio de Janeiro 21941-906, Brazil; babinayane@gmail.com (B.N.R.F.S.); betebergmaria@gmail.com (E.R.F.R.R.); alinedbcg@gmail.com (A.O.d.S.d.B.); msopijeira@gmail.com (M.S.O.P.); 2 Laboratory of Nanoradiopharmaceuticals and Synthesis of Novel Radiopharmaceuticals, Nuclear Engineering Institute, Brazilian Nuclear Energy Commission, Rio de Janeiro 21941-906, Brazil; hkenup@ien.gov.br; 3 DEFARMED Laboratory, Faculdade de Farmácia, Universidade Federal do Rio de Janeiro, Rio de Janeiro 21941-900, Brazil; ricci@pharma.ufrj.br; 4 Laboratory of Biophysics and Nanosystems, Department of Physics, Federal University of Maranhão, São Luís 65080-805, Brazil; joelfelixdiniz@gmail.com (J.F.S.D.F.); cleniltoncs@gmail.com (C.C.d.S.); lucianamagal@gmail.com (L.M.R.A.); 5 Center for Nanotechnology in Drug Delivery, Eshelman School of Pharmacy, Division of Pharmacoengineering and Molecular Pharmaceutics, University of North Carolina at Chapel Hill, Chapel Hill, NC 27599, USA; mattia@email.unc.edu; 6 Instituto de Ciências Biomédicas, Universidade Federal do Rio de Janeiro, Rio de Janeiro 21941-902, Brazil; sara.gemini@hotmail.com; 7 Laboratory of Radiopharmacy and Nanoradiopharmaceuticals, Zona Oeste State University, Rio de Janeiro 23070-200, Brazil

**Keywords:** alpha–beta therapy, bone cancer, radium-223 dichloride, radioactive gold nanoparticles, nanomicelles

## Abstract

Alpha and beta particulate radiation are used for non-treated neoplasia, due to their ability to reach and remain in tumor sites. Radium-223 (^223^Ra), an alpha emitter, promotes localized cytotoxic effects, while radioactive gold (^198^Au), beta-type energy, reduces radiation in the surrounding tissues. Nanotechnology, including several radioactive nanoparticles, can be safely and effectively used in cancer treatment. In this context, this study aims to analyze the antitumoral effects of [^223^Ra]Ra nanomicelles co-loaded with radioactive gold nanoparticles ([^198^Au]AuNPs). For this, we synthesize and characterize nanomicelles, as well as analyze some parameters, such as particle size, radioactivity emission, dynamic light scattering, and microscopic atomic force. [^223^Ra]Ra nanomicelles co-loaded with [^198^Au]AuNPs, with simultaneous alpha and beta emission, showed no instability, a mean particle size of 296 nm, and a PDI of 0.201 (±0.096). Furthermore, nanomicelles were tested in an in vitro cytotoxicity assay. We observed a significant increase in tumor cell death using combined alpha and beta therapy in the same formulation, compared with these components used alone. Together, these results show, for the first time, an efficient association between alpha and beta therapies, which could become a promising tool in the control of tumor progression.

## 1. Introduction

Targeted radionuclide therapy consists of a modality of treatment in which a biological effect is obtained by the energy absorbed from the radiation emitted by the radionuclide. Targeted radionuclide therapy is an optimal choice for intractable tumors [1]. There are the following three types of particulate radiation used for targeted radionuclide therapy: (i) alpha particles, (ii) beta particles, and (iii) Auger electrons. According to Ersahin et al. [2], radionuclides that emit α- or β-particles are preferred for the treatment of bulky tumors. At the same time, Auger electrons can be used for small clusters of cancer cells or small tumor deposits. For therapeutic purposes, radiations with high linear energy transfer (LET), such as α- and β-particles, are preferable [3].

The efficacy of systemic or localized cancer therapy rests on the ability of the radionuclide to reach and remain in the tumor site specifically. In this regard, to increase the targeting and efficacy of radionuclide therapy, the use of these radionuclides, inserted into nanoplatforms, may represent an innovative and effective approach, since nanoparticles (NPs) may (i) improve the bioavailability, (ii) increase the biological half-life, and (iii) increase the targeting of the drug to a specific location [4,5,6,7,8].

^198^Au is a beta-emitting radioisotope that has attracted interest in cancer molecular therapy using radioactive nanoparticles [9]. The mean penetration range (0.38 mm) in the tissue of beta particles of ^198^Au (0.960 MeV_max_) is sufficient to destroy tumor cells [10]. The beta particles have less energy than alpha particles; consequently, they cause less cytotoxicity. However, they can travel longer distances in circulation and damage DNA, promoting tumor cell death [11,12]. Moreover, ^198^Au has an appropriate half-life (2.7 d) for clinical application [10]. Thus, the conversion of ^198^Au into [^198^Au]AuNPs is an attractive option, due to its ability to produce varying forms and sizes, enabling direct delivery to the cancer site [13,14].

During the last decades, gold nanoparticles (AuNPs) have exhibited promissory potential for the diagnosis of various cancers and treatment, owing to their morphological and structural nature, surface chemical modification, and tunable sizes and shapes [15,16,17,18,19,20,21]. Gold is considered the most stable nanoparticle and the most common among noble metals [22,23]. It has low cytotoxicity compared to other metals, high targeting ability, well-established synthesis, easy surface functionalization, and high bio-interaction with target cells, and is non-immunogenic [16,22,24,25,26,27,28,29,30]. Furthermore, AuNPs can be prepared in different shapes, such as sticks, cages, and spheres, with sizes ranging from 1 nm to over 100 nm [31]. Conversely, [^198^Au]AuNPs are of increasing interest for local radionuclide therapy, as a powerful alternative for cancer treatment [15,32,33,34,35]. Several reports have shown the excellent efficacy of [^198^Au]AuNPs for tumor shrinkage in vivo [14,36,37,38]. Furthermore, [^198^Au]AuNPs have the advantage of being nanosized radioactive particles, with the potential to contain several radioactive atoms in a single particle [39,40].

On the other hand, ^223^Ra is an alpha emitter radionuclide that emits high-energy alpha particles (95.3% (energy range of 5.0–7.5 MeV)) of short range (<100 μm/10 μm cell diameters) [41,42,43,44], which endows short penetration, promoting localized cytotoxic effects, with shallow toxic effects on the adjacent healthy tissue [45,46,47]. In addition, it is a radionuclide with a suitable half-life (11.4 d) for clinical application [19,48]. The ^223^Ra acts as a calcium mimetic drug, predominantly accumulating in the bone tissues of the body [48,49]. In fact, ^223^Ra is currently used in targeted alpha therapy (TAT) for treating bone metastases of patients with metastatic castration-resistant prostate cancer [50]. For this, ^223^Ra is used in the chemical form of the salt radium dichloride ([^223^Ra]RaCl_2_). However, the preparation of stable formulations of ^223^Ra is challenging because of the daughter isotopes’ recoil energy and different chemical properties [51]. Although there are carriers reported for ^223^Ra, such as liposomes [52], iron oxide [53], hydroxyapatite particles [54], etc., there is still a problem in the radionuclide formulation for generating a stable product, with targeted distribution to organs and tissues [48]. In this sense, the use of 127-Pluronic nanomicelles works as an alternative to solve this problem.

Nanomicelles are well known as excellent drug delivery systems; they have structural stability, nanoscale size, and low cytotoxicity, and they minimize drug degradation, reduce adverse side effects, and improve drug bioavailability [55,56]. Nanomicelles are colloidal structures (5–100 nm), formed from amphiphilic monomers, with a hydrophilic outer layer and a hydrophobic inner layer. Due to their amphiphilic nature, normal nanomicelles encapsulate hydrophobic drugs and aid in their delivery. In the case of reverse nanomicelles, they can be used to encapsulate hydrophilic drugs, acting as better candidates for their delivery [57,58]. However, only a few works have reported the use of nanomicelles for the delivery of therapeutic radionuclides. These radionuclides were yttrium-90 and iodine-131 [59,60], which decay by the emission of beta particles. Thus, there are a lack of nanomicelles radiolabeled with alpha radionuclides, such as ^223^Ra.

Recently, our group developed 127-Pluronic-[^223^Ra]RaCl_2_ nanomicelles, which showed dose–response behavior and an increased effect on osteosarcoma cells, decreasing the cell viability more efficiently [61]. Pluronic F127 is a poly(ethylene oxide)–poly(propylene oxide)–poly(ethylene oxide) (PEO–PPO–PEO) copolymer, which forms micelles with the PPO as a hydrophobic core and the terminal PEO segment acting as a hydrophilic corona [62,63,64]. Polymeric micelles, such as Pluronic^®^, have often been employed for sustained release, achieving an extended circulation time, favorable biodistribution, reduced side effects, and lower toxicity [55,65]. Nanomicelles, compared with conventional micelles, are more thermodynamically stable in physiological solutions [57]. Moreover, nanomicelles show some advantages, due to their great biocompatibility, simple preparation methods, effectiveness, and low cost [66].

Therefore, the need to modernize antitumor therapies has led to nanotechnology aiming to develop a more effective, safe, and efficient therapy. In this sense, radioactive nanoparticles have gained attention, due to their physical-chemical characteristics, biocompatibility, low toxicity, bioconjugation, and few side effects for healthy cells [67,68,69]. Although the literature states that alpha therapy is superior to beta therapy [1], there is a total absence of combined alpha and beta therapy for tumor treatment.

This study has developed a nano-formulation of [^223^Ra] RaCl_2_ co-loaded with [^198^Au] Au nanoparticles for bone cancer therapy.

## 2. Materials and Methods

### 2.1. Reagents

Phosphate-buffered saline (PBS), PBS/EDTA, bovine serum albumin (BSA), methylated bovine serum albumin (mBSA), Freund’s complete adjuvant, Histopaque reagent, Pluronic F127, TRAcP staining kit, DMEM high glucose, fetal bovine serum, M-CSF, RANK-L, doxorubicin, chloroauric acid, tetraoctylammonium bromide, sodium borohydride, Poly-D-lysine, glucose, HEPES, calcium, and magnesium were purchased from Sigma Aldrich (St. Louis, MO, USA).

### 2.2. Preparation of the Nano-Formulations

#### 2.2.1. [198. Au]AuNPs

Firstly, the non-radioactive AuNPs were synthetized. A gold solution of chloroauric acid (HAuCl_4_ × 3H_2_O, 0.20 mmol, 78.7 mg) and tetraoctylammonium bromide (TOABr, 0.23 mmol, 126.8 mg) was dissolved in 10 mL of methanol and stirred vigorously for 24 h. Then, sodium borohydride (NaBH_4_ 2 mmol, 75.6 mg, dissolved in 5 mL of ice water) was added to the mixture, and the solution was kept under stirring for 8 h. After this period, the reaction mixture was ultracentrifuged (40,000 rpm for 30 min) to remove insoluble agglomerates. The supernatant was collected and concentrated by evaporation. The agglomerates were precipitated by adding ethanol to the solution. Then, the precipitate was extracted with minimal amounts of methanol several times. The AuNPs solution was precipitated again by ethanol and finally dried under a vacuum.

Then, the samples of AuNPs were irradiated in the Argonauta reactor (power of 340 W), installed at the Nuclear Engineering Institute (Brazil). The sample was irradiated for 12 h using a thermal neutron flow of 3.2 × 109 n·cm^−2^·s^−1^, with an average thermal neutron energy of 0.0025 eV.

#### 2.2.2. Radioactivity Measure

The induced activity of the [^198^Au]AuNPs was determined by a gamma spectrometry system with a hyper pure germanium (HPGe) detector, with a diameter of 6.2 cm, height of 4 cm, active volume of 41.1 cm^3^, and detection efficiency of 30%, coupled with the multichannel analyzer (Canberra) with 8192 channels. The detector was surrounded by a lead cover of ~10 cm to reduce the background. The measurement time for each sample was standardized at 3600 s (1 h).

### 2.3. Detection Efficiency

The detection efficiency for each energy type was determined using a LabSOCS (Laboratory SOurceless Calibration Software, Canberra, Australia). It was necessary to design the geometry used in a computational environment by inserting the physical, chemical, and geometric characteristics of the sample holder used, the detector, and the sample to be analyzed. After entering the data, the software simulates the detection efficiency values for each energy type. Then, the software doubles the number of voxels and repeats the entire process, obeying the convergence criteria and comparing the values until satisfactory convergence is obtained.

#### 2.3.1. Nanomicelles of [^223^Ra]RaCl_2_

A mass of 1 mg/mL of [^223^Ra]RaCl_2_ was weighed and added to the micellar dispersion of Pluronic F127. The system was gently stirred using a magnetic bar (Magnetic Stirrer, IKA, C-MAG HS-7) for 5 min and then processed for 5 min using an ultrasonic processor (UP100H, Hielscher, power: 100%, cycle: 1) in an ice bath at 10 °C.

#### 2.3.2. Nanomicelles of [^223^Ra]RaCl_2_ Co-Loaded with [^198^Au]AuNPs

A mass of 1 mg/mL of [^223^Ra]RaCl_2_ (~3.7 MBq) was weighed and added to the micellar dispersion of Pluronic F127. The system was gently stirred using a magnetic bar (Magnetic Stirrer, IKA, C-MAG HS-7) for 5 min and then processed for 3 min using an ultrasonic processor (UP100H, Hielscher, power: 100%, cycle: 1) in an ice bath at 10 °C. Then, a mass of 500 µg of [^198^Au]AuNPs (~1.85 MBq) was added and ultrasonicated for more than 2 min using an ultrasonic processor (UP100H, Hielscher, power: 100%, cycle: 1) in an ice bath at 10 °C.

The dispersion of polymeric nanomicelles containing [^223^Ra]RaCl_2_ and [^198^Au]AuNPs was stored in an amber flask for further analysis, in refrigeration (2–8 °C).

### 2.4. Characterization

#### 2.4.1. Particle Size

The particle size, size distribution, and polydispersity index (PDI) of the [^198^Au]AuNPs, 127-Pluronic-[^223^Ra]RaCl_2_ nanomicelles, and nanomicelles of [^223^Ra]RaCl_2_ co-loaded with [^198^Au]AuNPs were determined by dynamic light scattering (DLS), using Zetasizer Nano ZS (Malvern Instruments, Malvern, UK). Measurements were performed in triplicate at 25 °C, and the laser incidence angle in relation to the sample was 173° using a 12 mm^2^ quartz cuvette. The mean ± standard deviation (SD) was assessed.

#### 2.4.2. Atomic Force Microscopy

The AFM analysis was performed using a Multimode 8 microscope (Bruker, Santa Barbara, CA, USA). The following three central characterizations were conducted: [^198^Au]AuNPs, 127-Pluronic-[^223^Ra]RaCl_2_ nanomicelles, and nanomicelles of [^223^Ra]RaCl_2_ co-loaded with [^198^Au]AuNPs. ScanAsyst-Air probes (Bruker, Santa Barbara, CA, USA) were used for these measurements, with a nominal tip ratio of 2 nm and a nominal spring constant of 0.4 N/m. However, the actual spring constant was calibrated by the thermal noise method. A drop of all sample solutions was deposited in freshly cleaved mica. The scanning mode used was PeakForce Tapping Quantitative Nanomechanics (QNM), with a resolution of 256 × 256 lines per scan and a scan frequency of 0.5 Hz.

### 2.5. In Vitro Cytotoxicity

#### 2.5.1. Cell Culture

The SaOS-2 cells, a human osteosarcoma cell line, were plated in a density of 1 × 10^4^ cells/well for 24h. The cells were maintained in a DMEM/D-glucose (high glucose) medium, supplemented with 10% FBS, penicillin (0.5 U/mL), and streptomycin (0.5 mg/mL). The cells were incubated at 37 °C in a humidified atmosphere of 5% CO_2_. The cells were grown to confluence in 75 cm^2^ culture flasks and were detached by brief treatment with trypsin (0.1%)/EDTA (0.01%).

#### 2.5.2. Proliferation Assay

The SaOS-2 cells (1 × 10^4^ cells/well) were seeded and allowed to attach for 24 h. The cells were divided into the following three groups: pure [^223^Ra]RaCl_2_, 127-Pluronic-[^223^Ra]RaCl_2_+[^198^Au]AuNPs nanomicelles, and 127-Pluronic-[^223^Ra]RaCl_2_ nanomicelles, in the following three distinct activities: C1: 37 kBq, C2: 18.5 kBq, and C3: 4.44 kBq. The radioactivity ratio of ^223^Ra to ^198^Au in the co-loaded nanomicelles is 8.4. After 24 h, the cells were washed, and the number of attached cells was determined using the MTT assay.

#### 2.5.3. Statistical Analysis

Statistical analysis of the data was performed using the GraphPad Prism 7.3 software (GraphPad Software, San Diego, CA, USA). The differences between the means of the two groups were compared using the one-way ANOVA test and confirmed by the Bonferroni post-test. The results are presented as means ± standard deviation (S.D.). The values of * *p* < 0.05, ** *p* < 0.01, *** *p* < 0.005, and **** *p* < 0.0001 will be considered statistically significant.

## 3. Results

### 3.1. Synthesis and Irradiation of Gold Nanoparticles (AuNPs)

**Figure 1:**
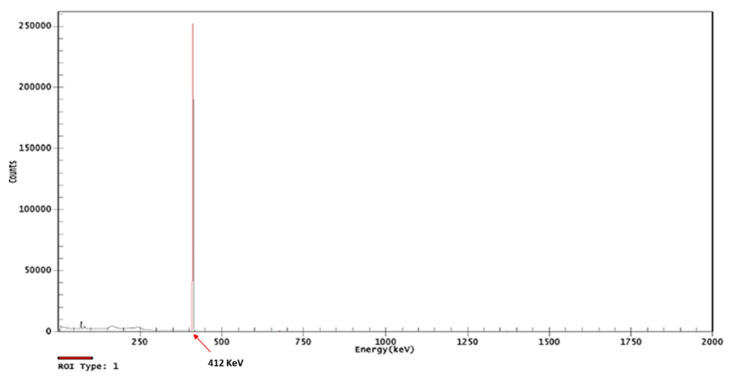
Gamma spectrometry using an HPGe detector from the ^198^Au, corroborating the efficacy of the irradiation process in forming [^198^Au]AuNPs.

Once synthesized, the AuNPs were irradiated under pre-established conditions, and were then able to produce the [^198^Au]AuNPs. The gamma spectrum obtained with the HPGe detector shows the specific range (412 KeV) of ^198^Au, as shown in Figure 1.

### 3.2. Particle Size

**Figure 2:**
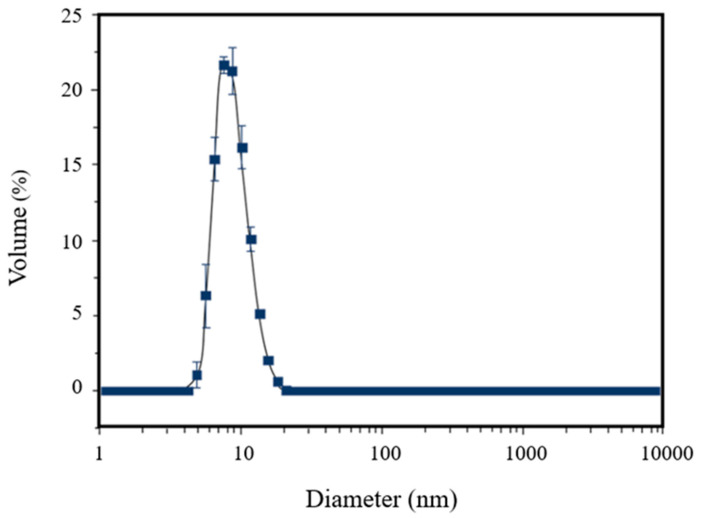
Dynamic light scattering analysis of [^198^Au]AuNPs, showing a mean size of 13 nm and a PDI of 0.106 (±0.089).

The DLS analysis of [^198^Au]AuNPs showed the formation of very small nanoparticles (13 nm) with very high monodisperse behavior, confirmed by the PDI (0.106) (Figure 2).

### 3.3. Atomic Force Microscopy

**Figure 3:**
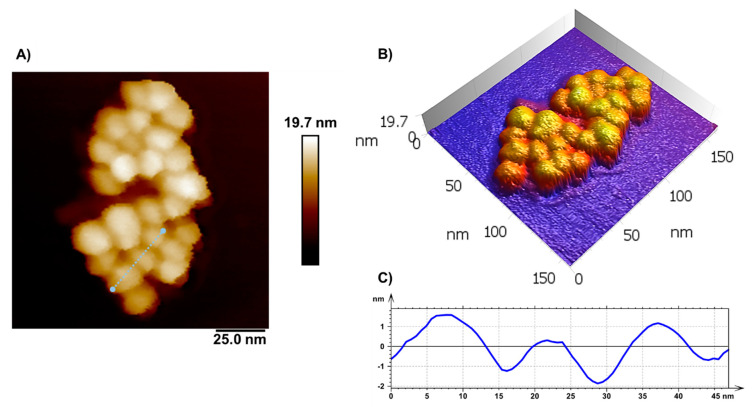
Topographic AFM image of [^198^Au]AuNPs. (**A**) Heightmap of AuNPs cluster and (**B**) its respective three-dimensional visualization. (**C**) Cross section over the AuNPs in the corresponding region, highlighted in image (**A**) (dotted line in light blue). The particle diameter observed in the AFM measurements is compatible with the values observed in the DLS measurements.

The morphology of the [^198^Au]AuNPs was investigated by AFM (Figure 3). Figure 3A shows an AFM height image of a cluster of [^198^Au] Au nanoparticles. The particles have a homogeneous morphology, with the maximum height on the map reaching 19.7 nm, in regions where the NPs are then superimposed, as evidenced in the three-dimensional image (Figure 3B). The cross section shown in Figure 3C corresponds to the three NPs marked with a dashed light blue line in Figure 3A. The diameters are 14.4 nm, 10.7 nm, and 13.1 nm, considering the horizontal distance taken from the width at the half-height of the particle. These values follow the DLS results.

### 3.4. Nanomicelles of [^223^Ra] RaCl_2_

#### 3.4.1. Particle Size

**Figure 4:**
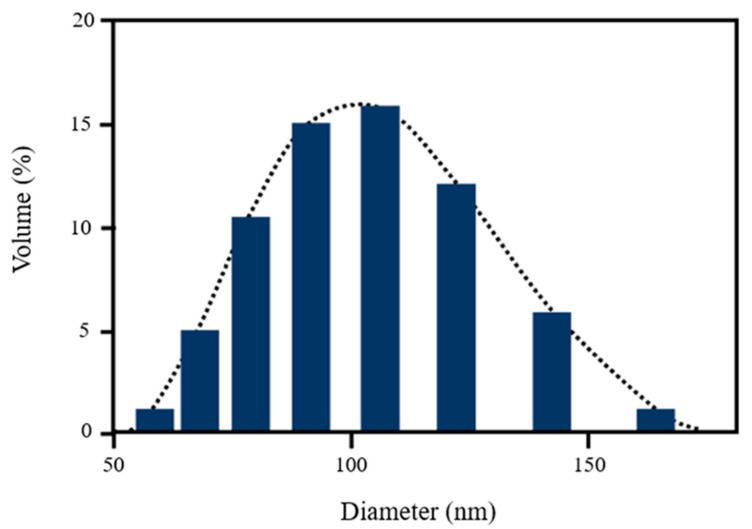
Dynamic light scattering analysis of [^223^Ra]RaCl_2_ nanomicelles, showing a mean size of 149 nm and a PDI of 0.0096 (±0.0002).

The dynamic light scattering analysis of [^223^Ra]RaCl_2_ nanomicelles showed a mean size of 149 nm, with a PDI of 0.0096 (±0.0002), corroborating the monodispersive value (Figure 4).

#### 3.4.2. Nanomicelles of [^223^Ra]RaCl_2_ Co-Loaded with [^198^Au]AuNPs

**Figure 5:**
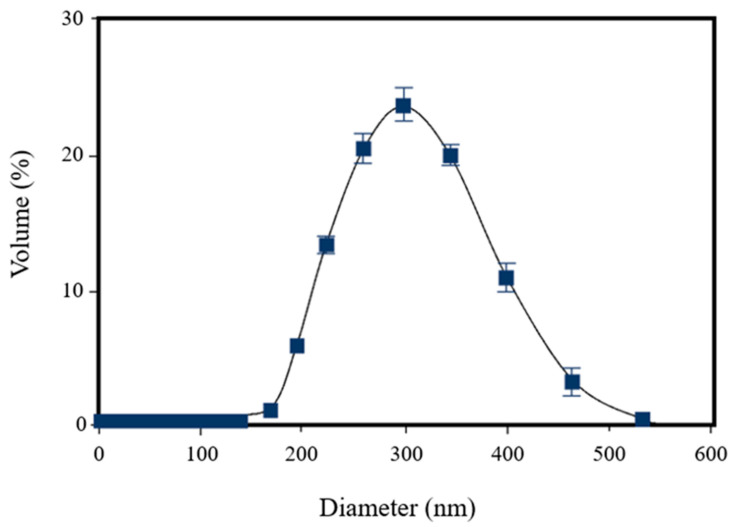
Dynamic light scattering analysis of [^223^Ra]RaCl_2_ co-loaded with [^198^Au]AuNPs, showing a mean size of 296 nm and a PDI of 0.201 (±0.096).

The DLS analysis of [^223^Ra]RaCl_2_ co-loaded with [^198^Au]AuNPs showed a larger size than the pure [^223^Ra]RaCl_2_, due to the incorporation of [^198^Au]AuNPs. The mean diameter was 296 nm. In addition, it was possible to observe an increase in the PDI value, probably due to the intermicellar destabilization caused by alpha and beta emission, as shown in Figure 5.

### 3.5. Atomic Force Microscopy of Nanomicelles Systems

**Figure 6:**
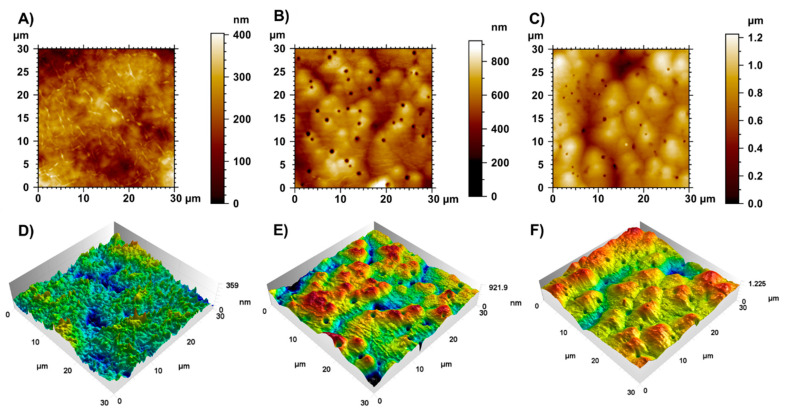
Atomic force microscopy height map of the pure (127-Pluronic) nanomicelles film (**A**), 127-Pluronic-[^223^Ra]RaCl_2_ nanomicelles film (**B**), and [^223^Ra]RaCl_2_ co-loaded with [^198^Au]AuNPs film (**C**), and their respective three-dimensional topographies (images (**D**–**F**)). The increase in height scale bar values indicates the filling of the 127-Pluronic nanomicelles with ^223^Ra and ^223^Ra + [^198^Au]AuNPs.

The analyses of the ultrastructure of pure nanomicelles (empty), [^223^Ra]RaCl_2_ nanomicelles, and [^223^Ra]RaCl_2_ co-loaded with [^198^Au]AuNPs nanomicelles were performed by AFM, and compared with the 127-Pluronic blank nanomicelles sample (Figure 6). Figure 6A shows a 127-Pluronic white nanomicelles film. Polymeric chain structures, with a diameter of 263.4 ± 12.1 nm, are observed. The maximum film height is 359 nm. The three-dimensional representation of Figure 6 is shown in Figure 6D–F, in which the absence of globular structures is evident.

Figure 6C shows the AFM height image of the [^223^Ra]RaCl_2_ co-loaded with [^198^Au]AuNPs film. Once again, it is possible to observe globular structures, with a maximum height of 1.225 μm. Such structures suggest the filling of 127-Pluronic nanomicelles with ^223^Ra and ^198^Au. Depressions in the micellar film are also present in this sample (Figure 6F). Since these holes are only observed in the 127-Pluronic-[^223^Ra] and [^223^Ra]RaCl_2_ co-loaded with [^198^Au]AuNPs films, they can be promoted by emitting alpha and beta particles from the radioactive nanomicelles. However, this emission is not able to destabilize the nanomicelles clusters.

### 3.6. In Vitro Cytotoxicity

**Figure 7:**
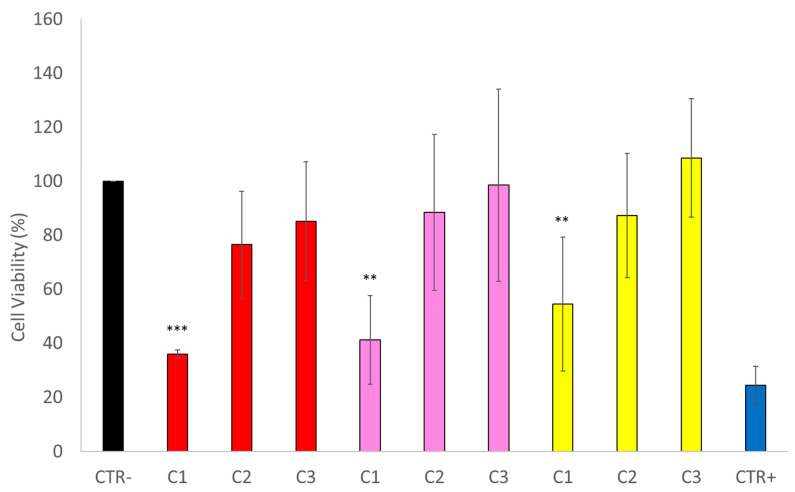
In vitro cytotoxicity results in human osteosarcoma SaOS-2 cells exposed to different nanosystems, using three different activities (C1: 37 kBq, C2: 18.5 kBq, and C3: 4.44 kBq) for each of the following formulations: [^223^Ra]RaCl_2_+[^198^Au]AuNPs nanomicelles (in red), [^223^Ra]RaCl_2_ nanomicelles (in pink), and [^223^Ra]RaCl_2_ (in yellow). ** *p* < 0.01, *** *p* < 0.005.

The cytotoxicity effect on human osteosarcoma of the nanosystems developed is expressed in Figure 7. It is possible to observe a very potent effect, mainly in 127-Pluronic-[^223^Ra]RaCl_2_+[^198^Au]AuNPs nanomicelles and 127-Pluronic-[^223^Ra]RaCl_2_ nanomicelles, when compared with the pure [^223^Ra]RaCl_2_, demonstrating that the combination of alpha and beta therapy increases the cytotoxicity effect.

## 4. Discussion

Radionuclide therapy is a safe and effective approach to treat primary cancers, as well as distant metastases. In this sense, beta- or alpha-emitting radionuclides have been primarily used [70]. However, there are a lack of radiopharmaceuticals that deliver simultaneous alpha and beta radiations, which would have remarkable potential for treating tumors. Alpha and beta particles have high energy in different magnitudes; consequently, they have different mean penetration ranges in tissue, for depositing their energies. Hence, alpha-/beta-labeled radiopharmaceuticals would combine the long-range crossfire effect of beta radiation with the DNA localization effect of alpha radiation, among other effects [1]. In addition, successful treatment would be reached using lower doses of alpha and beta emitters.

Therefore, we designed and prepared a novel nanoradiopharmaceutical, containing [^223^Ra]RaCl_2_ co-loaded with [^198^Au]AuNPs into nanomicelles, for radionuclide therapy of bone cancer using alpha and beta radiations simultaneously. Here, to the best of our knowledge, we presented the first findings of combining alpha and beta therapy in the same formulation. In addition, we are the first to report the radiolabeling of nanomicelles with ^223^Ra for delivering alpha radiation into tumor cells.

To fulfill our goal, the non-radioactive AuNPs were firstly fabricated using chemical synthesis. Through light scattering analysis, the AuNPs obtained a mean size of 13 nm and a PDI value of 0.106 (±0.089). Furthermore, the AFM analyses corroborated the DLS results, confirming the quality and homogeneity of our AuNPs. Then, the AuNPs were activated under neutron irradiation to obtain the [^198^Au]AuNPs, similar to previous reports [11]. Biodistribution studies (Table S1—Supplemental Information) have shown high liver uptake of [^198^Au]AuNPs at 2 h and 6 h post-injection in healthy mice. After 24 h of intravenous injection, the liver uptake decreased, while accumulating in the kidneys, suggesting renal clearance of [^198^Au]AuNPs.

Following this, the [^223^Ra]RaCl_2_ nanomicelles were prepared using a simple and fast method. The DLS analysis of [^223^Ra]RaCl_2_ nanomicelles showed a mean size of 149 nm and a PDI of 0.0096 (± 0.0002).

Finally, [^223^Ra]RaCl_2_ and [^198^Au]AuNPs were co-loaded into polymeric nanomicelles, and the final dispersion was characterized by DLS and AFM techniques. The DLS analysis revealed a mean size of 296 nm and a PDI value of 0.201 (±0.096). Moreover, the AFM images showed globular structures and holes because of the emissions of alpha and beta particles. Despite this, these emissions cannot destabilize the formed agglomerates, proving the quality of the nanomicelles.

Next, the in vitro cytotoxicity of these alpha–beta nanomicelles (127-Pluronic-[^223^Ra] RaCl_2_+[^198^Au]AuNPs) was evaluated in human osteosarcoma cells (SaOS-2). It was also compared with the in vitro cytotoxicity of the following two formulations: [^223^Ra]RaCl_2_ and 127-Pluronic- [^223^Ra]RaCl_2_. The results showed that the three formulations could kill SaOS-2 cells using three activities (37 kBq, 18.5 kBq, and 4.44 kBq). Nevertheless, the 127-Pluronic-[^223^Ra]RaCl_2_+[^198^Au]AuNPs formulation significantly increased cell death as the radioactive activity increased (37 kBq), compared with other activities and formulations. Hence, these findings are very promising. Radionuclide therapy has the advantage of delivering a concentrated dose to target tumor tissues, while preserving the surrounding healthy tissues, unlike the other current cancer therapies [19,39,67,71,72]. For the first time, the simultaneous ability of alpha and beta radiations to kill cancer cells has been demonstrated, using a low radioactive dose and obtaining high efficacy.

## 5. Conclusions

The combination of alpha and beta radiations in the same nanoprobe would represent a very efficient tool for cancer treatment. Our in vitro findings are the first to demonstrate the remarkable ability of alpha–beta nanoprobes to kill cancer cells using a low radioactive dose. Future works should aim to evaluate the in vivo therapeutic effect and safe use of our nanomicelles of [^223^Ra]RaCl_2_ co-loaded with [^198^Au]AuNPs in bone cancer-bearing mice. Conversely, this work may lead to further studies involving the functionalization of these alpha–beta nanoprobes for targeted radionuclide therapy beyond bone cancer.

## Data Availability

All data will be available under request.

## Supplementary Materials

The following supporting information can be downloaded at: https://www.mdpi.com/article/10.3390/polym14071405/s1, Table S1: Biodistribution data of [^198^Au]AuNPs in healthy mice.
