# Supplementary material for: Nanomicelles of Radium Dichloride [223Ra]RaCl2 Co-Loaded with Radioactive Gold [198Au]Au Nanoparticles for Targeted Alpha–Beta Radionuclide Therapy of Osteosarcoma
Source: Polymers (Basel). 2022 Mar 30;14(7):1405. doi: 10.3390/polym14071405 (PMC9002948; doi:10.3390/polym14071405)
Supplement: Supplementary file 1 [file polymers-14-01405-s001.zip › polymers-1657057-supplementary.pdf]

polymers

MDPI

Supplementary Materials: Nanomicelles of Radium Dichloride [^{223}Ra]$RaCl_2$ Co-Loaded with Radioactive Gold Nanoparticles [^{198}Au] Au-Nanoparticles for Targeted Alpha-Beta Radionuclide Therapy of Osteosarcoma

Bárbara Nayane Rosário Fernandes Souza, Elisabete Regina Fernandes Ramos Ribeiro, Aline Oliveira da Silva de Barros, Martha Sahylí Ortega Pijeira, Hericka Oliveira Kenup-Hernandes, Eduardo Ricci-Junior, Joel Félix Silva Diniz Filho, Clenilton Costa dos Santos, Luciana Magalhães Rebelo Alencar, Mohamed F. Attia, Sara Gemini Piperni and Ralph Santos-Oliveira

Citation: Souza, B.N.R.F.; Ribeiro, E.R.F.R.; da Silva de Barros, A.O.; Pijeira, M.S.O.; Kenup-Hernandes, H.O.; Ricci-Junior, E.; Diniz Filho, J.F.S.; dos Santos, C.C.; Alencar, L.M.R.; Attia, M.F.; et al. Nanomicelles of Radium Dichloride [^{223}Ra]$RaCl_2$ Co-Loaded with Radioactive Gold [^{198}Au]Au Nanoparticles for Targeted Alpha–Beta Radionuclide Therapy of Osteosarcoma. *Polymers* **2022**, *14*, 1405. https://doi.org/10.3390/polym14071405

Academic Editor: Dimitrios Bikiaris

Received: 13 March 2022
Accepted: 26 March 2022
Published: 30 March 2022

Publisher's Note: MDPI stays neutral with regard to jurisdictional claims in published maps and institutional affiliations.

Copyright: © 2022 by the authors. Licensee MDPI, Basel, Switzerland. This article is an open access article distributed under the terms and conditions of the Creative Commons Attribution (CC BY) license (http://creativecommons.org/licenses/by/4.0/).

Biodistribution of [^{198}Au]AuNPs in healthy mice

Materials and methods

Animals

Naïve C57BL/6 male mice (n = 6, 20–30 g), were obtained from Oswaldo Cruz Foundation breeding unit (Rio de Janeiro, Brazil) and housed in standard plastic cages coated with white Pinus wood shavings as bedding, and stainless-steel cover lids. Cages were maintained under controlled room temperature (23 ± 2 °C), relative air humidity (app. 70%), and a 12 h light-dark cycle. All mice were kept with free access to filtered water and standard rodent chow (ad libitum). All procedures were approved by the Zona Oeste State University (CEUA UEZO Nº 8059100220.) which is consistent with United States National Institute of Health Guide for Care and Use of Laboratory Animals (National Research Council, 1996).

Animal Preparation and Injection

Animals were anesthetized with isoflurane (2–3%) and kept warmed in individual cages. The [^{198}Au] Au-Nanoparticles were injected by retro-orbital (R.O.) via in a concentration of 3.7 MBq in 0.2 mL. All animals were euthanized by CO_2 inhalation.

Biodistribution

Biodistribution analysis was performed by collecting blood and organ samples (heart, brain, stomach, pancreas, small and large intestine, bladder, kidneys, lungs, liver, and spleen) from C57BL / 6 mice (naive, n = 6) at three different times (2, 6 and 24 h).The blood and organs collected were weighed, and each organ and blood activity was counted by a gamma counter (Perkin Elmer Wizard®). The results were expressed as dose per organ (% DI/organ) and dose per gram of tissue (% DI/g).

Results

The results (Table S1) showed that a high amount of [^{198}Au]AuNPs were uptake by the liver during the first 2 h (98.29%) and 6 h (98.28%). In 24h post-injection, a reduction in liver uptake was followed by an increased presence in kidneys, demonstrating a renal clearance.

Table S1. Biodistribution data of [^{198}Au]AuNPs in healthy mice.

**Organ/Tissue**	**% DI/g**		
	**2 h**	**6 h**	**24 h**
Blood	0.00	0.01	0.04
Heart	0.00	0.01	0.02
Brain	0.00	0.01	0.03
Stomach	0.01	0.01	0.05
Pancreas	0.00	0.01	0.03
Small Ints.	0.01	0.04	0.04
Large Ints.	0.01	0.01	0.02
Bladder	0.02	0.45	0.01
Left Kidney	0.01	0.03	11.27
Right Kidney	0.01	0.04	6.31
Left Lung	0.20	0.21	0.13
Right Lung	0.15	0.21	0.25
Liver	98.29	98.28	84.39
Spleen	1.40	0.69	1.04
